# Initial Biological Assessment of Upconversion Nanohybrids

**DOI:** 10.3390/biomedicines9101419

**Published:** 2021-10-09

**Authors:** Juan Ferrera-González, Laura Francés-Soriano, Cristina Galiana-Roselló, Jorge González-Garcia, María González-Béjar, Eleonore Fröhlich, Julia Pérez-Prieto

**Affiliations:** 1 Instituto de Ciencia Molecular (ICMol), Departamento de Química Orgánica, University of Valencia, Catedrático José Beltrán, 2, Paterna, 46980 Valencia, Spain; juan.ferrera@uv.es (J.F.-G.); laura.frances-soriano@univ-rouen.fr (L.F.-S.); cristina.galiana@uv.es (C.G.-R.); jorge.gonzalez@uv.es (J.G.-G.); 2 nanoFRET.com, Laboratoire COBRA (Chimie Organique, Bioorganique: Réactivité et Analyse), UMR 6014, CNRS, Université de Rouen Normandie, INSA, CEDEX, 76821 Mont-Saint-Aignan, France; 3 Center for Medical Research, Medical University of Graz, Stiftingtalstr. 24, 8010 Graz, Austria

**Keywords:** upconversion nanoparticles, cucurbituril, cytotoxicity

## Abstract

Nanoparticles for medical use should be non-cytotoxic and free of bacterial contamination. Upconversion nanoparticles (UCNPs) coated with cucurbit[7]uril (CB[7]) made by combining UCNPs free of oleic acid, here termed bare UCNPs (UC_n_), and CB[7], i.e., UC@CB[7] nanohybrids, could be used as photoactive inorganic-organic hybrid scaffolds for biological applications. UCNPs, in general, are not considered to be highly toxic materials, but the release of fluorides and lanthanides upon their dissolution may cause cytotoxicity. To identify potential adverse effects of the nanoparticles, dehydrogenase activity of endothelial cells, exposed to various concentrations of the UCNPs, was determined. Data were verified by measuring lactate dehydrogenase release as the indicator of loss of plasma membrane integrity, which indicates necrotic cell death. This assay, in combination with calcein AM/Ethidium homodimer-1 staining, identified induction of apoptosis as main mode of cell death for both particles. The data showed that the UCNPs are not cytotoxic to endothelial cells, and the samples did not contain endotoxin contamination. Higher cytotoxicity, however, was seen in HeLa and RAW 264.7 cells. This may be explained by differences in lysosome content and particle uptake rate. Internalization of UC_n_ and UC@CB[7] nanohybrids by cells was demonstrated by NIR laser scanning microscopy.

## 1. Introduction

The combination of macrocyclic hosts and photoactive inorganic nanomaterials allows for the design of new functional photoactive inorganic–organic hybrid nanomaterials that benefit from the properties of the individual counterparts [1]. Our interest in this communication focuses on photoactive nanohybrids which combine recognition based on host–guest chemistry and the unique photophysical properties of photoactive nanoparticles [2]. Macrocycles, such as crown ether, cyclodextrins, calixarenes, cucurbiturils, and pillarenes, do not only confer hydrophilicity to the resulting nanohybrids, but also preserve their supramolecular recognition capacity for subsequent self-assembly when acting as surface capping agents for photoactive inorganic nanoparticles [2]. Moreover, these nanohybrids could be used as stimuli-responsive smart nanocarriers to deliver a selected encapsulated cargo [3,4].

In this context, upconversion nanoparticles (UCNPs) are suitable for use in bioapplications, such as imaging, sensing, drug delivery, and therapy [5]. UCNPs are composed of an inorganic matrix doped with photoactive lanthanide cations that can be excited with low-energy near-infrared (NIR) photons (two or more) and emit at shorter wavelengths (ultraviolet, visible light, and NIR), thus giving rise to anti-Stokes emission and highly intensive narrow emission bands at wavelengths which depend on the selected lanthanides. Remarkably, UCNPs present good photochemical, chemical, and thermal stability.

Moreover, due to their capacity to absorb NIR excitation, the autofluorescence from biological samples and scattered excitation light are eliminated, while deep tissue penetration is accomplished (when compared to shorter excitation wavelengths). Thus, good signal-to-background ratios and high sensitivities are achieved. Additionally, UCNPs are very resistant to photo-bleaching and photo-blinking and present relatively low (cyto)toxicity [6,7].

Typically, UCNPs are synthesized in hydrophobic solvents and are capped with oleate (OA), which anchors to the UCNP surface via the carboxylate group generated in basic conditions [8]. The alkyl chain of the ligand confers them dispersibility in organic solvents. However, the use of UCNPs in biological applications, such as therapy, imaging, and biosensing, requires their post-synthetic surface modification with biocompatible and water-soluble ligands. The molecular recognition capacity of water-soluble macrocycles makes them promising surface ligands for the functionalization and further bioapplication of UCNPs.

Cyclodextrins are cyclic oligosaccharides which contain a doughnut-shaped structure (α-, β-, and γ-CD) and have received considerable attention for UCNP post-synthetic surface modification to provide them water-dispersibility while retaining the molecular recognizing capacity of the macrocyclic capping [2,9]. For example, the simple phase transfer of OA-stabilized UCNPs from organic phase to water medium has been conducted with γ-CD and α-CD by forming host-guest complexes with OA [10,11].

Interestingly, CDs are relatively easy to modify chemically, and they can be designed to enable simple conjugation with various functional molecules, such as organic dyes and biomolecules [9,10,12,13]. UCNPs have been coated with carboxymethyl-β-cyclodextrin (COOH-β-CD) to serve as host to connect adamantine modified phthalocyanine (Ad-ZnPc) as guest of COOH-β-CD (K_a_ = 1500 K M^−1^ for adamantine with β-CD), thus permitting a short-distance linking between the UCNPs and the Ad-ZnPc photosensitizer. This nanohybrid presented a strong NIR light-triggered PDT activity toward cancer cells [12].

In addition, UCNPs modified with 6-phosphate-6-deoxy-β-cyclodextrin (β-PCD) showed a superior stability and cell viability (>90% for 200 μg/mL) in comparison to their covering with silicon dioxide or poly(acrylic acid) [9]. Additionally, the addition of a β-CD outer layer to the periphery of UCNPs covered with a shell of mesoporous SiO_2_ (mSiO_2_) loaded with a rhodamine B (RdB)-derived molecules (RdMs), led to an improvement of the stability and biocompatibility of the nanosystem [14], besides from preventing RdB leaching and blocking large molecules from entering the mesoporous channels while remaining non-toxic to liver tissue slices obtained from mouse models and HeLa cells [14].

Cucurbit[n]urils (CB[n]) are water-soluble pumpkin-shaped rigid macrocyclic hosts made of glycoluril units linked by methylene (–CH_2_–) bridges that can cross the cell membrane [15]. Their diameter depends on the number of glycoluril units (n). Because of their hydrophobic cavity and the carbonyls groups at the edges, CB[n] can form strong host–guest inclusion complexes with charged organic compounds [16,17]. For example, amino acids have been used as guests for these host-guest interactions, thanks to the intermolecular hydrogen bonds that can form between the carbonyl groups and the amino acids [16]. CB[n] have no intrinsic cytotoxicity in many human cancer lines (IC_50_ > 100 mM, 50% of growth inhibition concentration) [18,19]. Therefore, CB[n] can be used in drug delivery, biomimetic systems, or photodynamic therapy. Moreover, CB[n] have higher binding association constants with guests than CDs, although CB[n] are more difficult to derivatize. 

The surface of UCNPs has been modified with cucurbit[7]uril (CB[7]) via supramolecular host–guest self-assembly, thus converting UCNPs from hydrophobic to hydrophilic [20]. Moreover, the CB[7] capping can enable further supramolecular conjugation of biomolecules via host–guest complexation [20]. 

We have coated bare NaYF_4_: Yb^3+^, Er^3+^ UCNPs with cucurbituril to lead to UC@CB[n] nanohybrids, and demonstrated the interaction between the surface of the UCNP and one of the carbonyl portals, using different cucurbit[n]urils (*n* = 6, 7, 8) [21]. The resulting UC@CB[n] nanohybrids displayed good water dispersibility and stability. The free carbonyl portal was able to interact with other molecules, such as dyes [21]. Although UCNPs are generally regarded as highly biocompatible nanoparticles, they may cause cytotoxicity by dissolution and release of fluoride and lanthanide ions [22]. To identify potential adverse cellular effects, UCNPs were tested on EAhy926 endothelial cells because intravenous injection is the common administration route of UCNPs and endothelial cells represent the lining cells of blood vessels. For parenteral administration, limits of contamination with endotoxin must be respected [23]. In the present work, the cytotoxicity and internalization of UC@CB[7] were also investigated in the cervical cancer HeLa cell line and the macrophage cell line RAW 264.7. RAW 264.7 is an established model for macrophages that actively and rapidly take up nanomaterials from biological media. This model has already been applied in studies involving bare NaGdF_4_ [24] and silica-coated UCNPs [25].

## 2. Materials and Methods

### 2.1. Chemicals 

Lanthanide chlorides (YCl_3_·6H_2_O, YbCl_3_·6H_2_O and ErCl_3_·6H_2_O (>99.9%, all of them)), 1-octadecene (95%), oleic acid (99.9%), NaOH, NH_4_F (99.99%), and cucurbit[7]uril were purchased from Sigma-Aldrich (Madrid, Spain) and used as received without previous purification.

### 2.2. Synthesis NaYF_4_: Yb^3+^(20%), Er^3+^(2%) Nanoparticles (UC_OA_)

Oleate-capped NaYF_4_: Yb, Er nanoparticles (UC_OA_) were synthesized by following a previously described protocol with some modifications [8]. In a 50 mL round-bottom flask, YCl_3_·6H_2_O (0.8 mmol), YbCl_3_·6H_2_O (0.20 mmol), and ErCl_3_·6H_2_O (0.02 mmol) were stirred at 160 °C in the presence of 12 mL of oleic acid and 15 mL of octadecene under a N_2_ flow. Once the lanthanide salts were completely dissolved, the mixture was allowed to cool to 110 °C. Then, 10 mL of a methanol solution containing NaOH (2.5 mmol) and NH_4_F (4.0 mmol) was slowly dropped into the flask. After methanol evaporation at 100 °C under N_2_, the reaction was heated at 305 °C under N_2_ for one hour. Finally, the solution was cooled down to room temperature and the nanoparticles were recovered by centrifugation (10000 rpm, 15 min, 25 °C). Later, the unreacted reagents were removed by washing three times the obtained white pellet UC_OA_ nanoparticles with a hexane/acetone/methanol (43.5:40.5:16 *v*/*v*) solution and once with ethanol.

### 2.3. Synthesis of Bare NaYF_4_:Yb, Er UCNPs (UC_n_) 

Oleate ligand was removed from the UCNPs surface by following a well-known procedure [26]. Briefly, 100 mg of UC@OA were stirred for 2 hours in 10 mL of HCl aqueous solution (pH = 4). The pH of the reaction was kept at 4 by the addition of an HCl solution (0.1 M). Then, the oleic acid was extracted from the reaction mixture with diethyl ether (×3) followed by the re-extraction with water of the combined organic phases. After that, the aqueous phases were combined and re-extracted with diethyl ether. Ligand-free UCNPs were precipitated by the addition of acetone and centrifugation (10,000 rpm, 15 min, 25 °C). The transparent precipitated was purified following three cycles of redispersion in acetone and centrifugation (10,000 rpm, 15 min, 25 °C). Finally, UC_n_ were dispersed in Milli Q water.

### 2.4. Functionalization of NaYF_4_:Yb, Er UCNPs with CB[7] (UC@CB[7]) 

CB[7] was attached to the ligand-free surface by following a previous protocol developed in our group [21]. A mixture containing 0.01 mmol of CB[7], 30 mg of UC_n_, and 15 mL of MilliQ water was sonicated for 15 minutes. The dispersion was stirred during 24 h in an orbital shaker at 350 ppm in the darkness. The resulting free CB[7] was removed from the solution with 2 cycles of sonication in 15 mL water (15 min) and centrifugation (10,000 rpm, 10 min, 25 °C) and 3 cycles in 15 mL of acetonitrile. The UC@CB[7] were redispersed in MilliQ water.

### 2.5. Upconversion Luminescence (UCL) Measurements

Steady-state UCL spectra of 10 mg/mL UCNP dispersions in dimethyl sulfoxide (DMSO) were recorded in a FLS1000 photoluminescence spectrometer (Edinburgh Instruments) coupled with a 2 W CW 980 nm laser diode (PSU-III-LED, CNI Optoelectronics Technology Co. Ltd., Changchun, China) at 2.3 W/cm^2^. The Fluoracle software was used to register the data.

### 2.6. High-Resolution Transmission Electron Microscopy (HRTEM)

HRTEM images were obtained using a Jeol 1010 TECNAI G2 F20 microscope operating at 200 kV (point resolution of 0.24 nm) and equipped with a CCD GATAN camera. For the preparation of the samples, 10 μL of a 0.5 mg/mL aqueous solution of the UCNPs were left to dry under vacuum at room temperature on a formvar/carbon film supported on a 300-mesh copper grid.

### 2.7. Zeta Potential

The zeta potential values were determined employing a Zetasizer Ultra (Malvern, UK). The measurements were carried out with a dispersion of 1 mg/mL in milliQ water.

### 2.8. Contamination with Bacteria and Endotoxin 

PYROGENT Ultra (sensitivity = 0.06 EU/mL, Szabo-Scandic, Vienna, Austria) was used for the endotoxin testing. Each sample dilution was tested in duplicate, and the different endotoxin standards with *E. coli* strain 055:B5 in triplicates. The assay is performed first as a yes/no test and samples with positive endotoxin detection can be further tested via a dilution series to quantify free endotoxin. The assay was performed according to the instructions given in the manual. 

### 2.9. Cell Exposures

Human macrovascular endothelial cells EAhy 926 were kindly provided by Dr. C.J. Edgell. The cells express typical features of endothelial cells, such as human factor VIII related antigen [27]. Cells were cultured in high glucose Dulbecco’s Modified Earls Medium (DMEM) supplemented with 10% fetal bovine serum (FBS), 2 mM L-glutamine, and 1% penicillin/streptomycin (GE Healthcare, Vienna, Austria) in a humidified atmosphere (37 °C, 5% CO_2_, 95% RH). Cell numbers were determined by a cell counter and analyzer system (CASY® TT, Innovatis, Vienna, Austria). For the experiments, cells (1.4 × 10^5^/well) were seeded in 96-well plates 24 h prior to the exposure to create subconfluent cultures (80% confluency) as recommended for viability testing according to the ISO 10993-5 guideline. UCNPs were tested in concentrations of 12.5, 25, 50, 100, and 200 μg/mL. To identify potential interference with the assays, particles alone were also tested. Then, 20 nm plain polystyrene nanoparticles (ThermoFisher Scientific, Vienna, Austria) in a concentration of 400 µg/mL were used as positive control. At this concentration, when applied in FBS-containing media, the viability of EAhy926 cells is expected to be 4–6% [28]. 

Cervical cancer HeLa and murine leukemia macrophage RAW264.7 cells from Central Service for Experimental Research (SCSIE) at University of Valencia were grown in high glucose phenol red-free Dulbecco’s modified Eagle medium (DMEM) containing 10% FBS, penicillin/streptomycin 1% and fungizone 0.01% at 37 °C with 5% CO_2_ in humidified atmosphere. Cells were kept continuously under confluence before split twice a week and the possibility of contamination was excluded by performing regular mycoplasma test.

### 2.10. Cellular Imaging

Cervical cancer HeLa and murine leukemia macrophage RAW 264.7 cells were seeded on culture dishes (*ca.* 5 × 10^5^, 10 mL, 10 cm^2^) for 24 h, then the media was replaced with fresh media containing UCNPs (50 μg/mL, 10 mL) for 24 h. Prior to imaging, the cells were washed with PBS and co-incubated with fresh growth media phenol red-free containing Hoechst 33342 (2 μM, 10 mL, 15 min). For fixation of the cells, upon nuclear staining with Hoechst, cells were incubated with paraformaldehyde 4% during 30 min, washed with PBS, and place fresh PBS before imaging. Cells were recorded on a commercial multi-photon excitation microscope (FV100MPE) comprising a BX61WI upright microscope with motorised focus and equipped with a XLPLN25 × WMP 1.05 NA (working distance 2 mm) full water immersion lens. The excitation source is a Mai-Tai HP Deep See (Spectra Physics) Ti-Sapphire pulsed tuneable laser with a range of 690 to 1040 nm and an average power of 2.0 W (pulse width: 100 fs; frequency: 80 MHz; peak power (800 nm): >266 kW) controlled by an acousto-optic modulator (AOM). The emission was detected via four photomultiplier tubes with specific filter cubes covering the whole visible range: detection channel 1 (C1), range 420 to 500 nm; channel 2 (C2), range 515 to 580 nm; channel 3 (C3), range 590 to 650 nm, and channel 4 (C4), range 660 to 740 nm. A computer-controlled imaging software (FV10-ASW) was used to select the laser excitation power and wavelength, and to acquire the emission images. UCNPs were excited at 975 nm and their emission collected in C2 (515–580 nm) at 100 μs/pixel dwell time (excitation fluence per dwell time: 220 J/cm^2^), while Hoechst 33342 was excited by two-photon absorption at 750 nm and its fluorescence was collected in C1 (420–500 nm) at 4 μs/pixel dwell time [29].

### 2.11. Dehydrogenase Activity (MTS) Assay

CellTiter 96® AQueous Non-Radioactive Cell Proliferation Assay (Promega, Mannheim, Germany) was used according to the manufacturer’s instruction. In short, 20 µL of the combined MTS/PMS solution was added to 100 µL of each well. Plates were incubated for 2 h at 37 °C in the cell incubator. Absorbance was read at 490 nm on a plate reader (SPECTRA MAX plus 384, Molecular Devices, Munich, Germany). Wells without cells but with the respective medium, in which the NPs were suspended, were used as blank control. To investigate whether UCNPs interfere with the assay, an interference control (=highest concentration of each UCNPs without cells) was included. Experiments were performed in triplicates.

### 2.12. Lactate Dehydrogenase (LDH) Release

The CytoTox-ONE™ Homogeneous Membrane Integrity Assay (Promega, Madison, WI, USA) was used according to the instructions given by the producer. In brief, 2 µL of Lysis solution (9% Triton X-100) was added to each lysis control well. At this concentration of the detergent, the plasma membrane is lysed but LDH activity is preserved. All wells received a volume of CytoTox-ONE reagent equal to the volume of cell culture medium present in each well. Incubation started after mixing for 30 s for 10 min at RT and was stopped by addition of 50 µL of stop solution (per 100 µL of CytoTox-ONE reagent added). Finally, the plate was shaken for 10 s and the fluorescence was recorded (FLUOstar Optima, servoLAB GmbH, Kumberg, Austria) at an excitation wavelength of 560 nm and an emission wavelength of 590 nm. After subtraction of the blank value, the average fluorescence from the samples was normalized to the maximum LDH release (lysis control). Experiments were performed in triplicates.

### 2.13. Cell Viability (MTT) Assay

The cytotoxic effects of UCNPs toward HeLa and RAW 264.7 cells were assessed by MTT assays for cell viability. The cells were seeded at a density of 5 × 10^4^ cells/mL (if we consider 5000 cells in 100 μL of culture media per well in 96-well plates). The culture medium was removed after the cells adhered to the wall, and they were treated with UCNPs at serial concentrations of 12.5, 25, 50, 100, and 200 μg/mL for 24 h. Then, the medium was removed, and the cells were washed with PBS. Finally, 90 μL of serum-free without red-phenol culture media and 10 μL MTT solution (5 mg/mL) were added to each well. After incubation for 4 h, the supernatant was removed and 100 μL DMSO was added to each well. The trays were then vigorously shaken to solubilize the formazan product and the absorbance at a wavelength of 490 nm was read on Microplate Reader Multiskan EX microplate reader (MTX Labsystems, Vantaa, Finland) and analyzed. All MTT assays were performed three times in duplicate. A negative control was also performed by exposing cells only to culture medium and a positive control was conducted by using 0.1% Triton X-100.

### 2.14. Calcein-AM and Ethidium Homodimer-1 (EthD-1) Staining 

Additionally, the cytotoxicity was assessed by means of the calcein AM/Ethidium homodimer-1 (EthD-1) staining assay (LIVE/DEAD^®^ Viability/Cytotoxicity Kit for mammalian cells, Molecular Probes, Inc., Eugene, OR). Briefly, HeLa and RAW 264.7 cells were cultured in 6-well plates and treated with the UCNPs at different concentrations for 24 h. Then, we collected together the supernatants and the cells detached by using trypsin 0.25%. The cell suspensions were centrifuged, the obtained pellets were suspended in 500 μL PBS and stained with 50 nM calcein AM (λ_ex_/λ_em_: 494/517 nm) and 16 µM EthD-1 (λ_ex_/λ_em_: 528/617 nm) for 30 min at 25 °C. Samples were analyzed using flow cytometry (BD FACSVerse, BD Biosciences, San Jose, CA, USA) at SCSIE (University of Valencia). According to Palma et al. [30], in the generated dot plots, EthD-1 positive and slightly negative calcein AM cells were considered dead, Calcein AM positive but EthD-1 negative cells as live, and the cells with either weakly or strong intensity of calcein AM and EthD-1 as apoptotic cells. 

## 3. Results

### 3.1. Synthesis of CB[7]-Capped UCNPs

**Figure 1:**
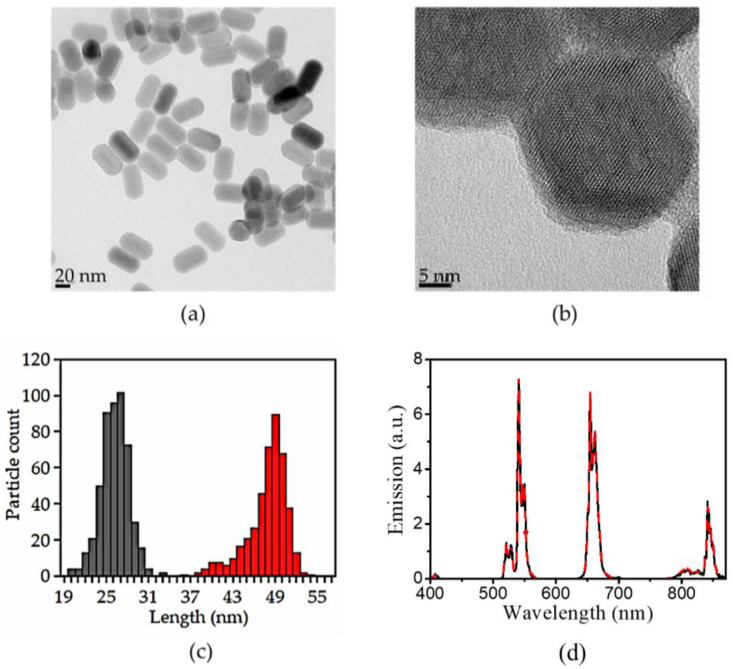
Representative HRTEM images of (**a**) UC_n_ and, (**b**) UC@CB[7] nanoparticles. (**c**) Histogram showing the size distribution of the NPs for the width (grey bars) and length (red bars). (**d**) Corrected emission spectra (λ_ex_ = 975 nm) of 10 mg/mL DMSO dispersions of ligand-free UCNPs (black line) and UC@CB[7] (red dotted line).

The functionalization of the UCNPs with CB[7] was achieved via a three-step synthesis previously developed in our group [21]. First, oleate-capped UCNPs were synthesized by the high-temperature co-precipitation method [8]. Then, the oleate ligand was removed by its protonation in acidic medium (pH = 4) for only 2 h [26]. The bare UCNPs were stable and no hydrolysis, corrosion, and aggregation were observed under mild acidic conditions (it must be pointed out that these inconvenient effects are observed in strong acidic media and in highly diluted samples) [31,32]. Then, CB[7] was allowed to coordinate to the ligand-free UCNP surface as a protective shell by simply stirring them together in an aqueous solution. Monodisperse (26.1 ± 1.9; 47.8 ± 1.9 nm) and highly crystalline nanohybrids were obtained as shown by HRTEM images (Figure 1). Energy-dispersive X-ray spectroscopy (EDAX, not shown) was used to elucidate the elemental composition of the synthesized NPs, obtaining an amount (at %) of the dopant lanthanides cations of 77.9 ± 0.48 for yttrium, 20 ± 3.0 for ytterbium, and 2.1 ± 0.9 for erbium. The thickness of the CB[7] organic layer was estimated to be *ca*. 1.5 nm.

Identical UCL spectra were registered upon NIR excitation of UC_n_ and UC@CB[7] due to the characteristics emissions of Er^3+^. The surface charge (zeta potential) of the UC_n_ dispersed in milliQ-water showed positive values (20 ± 2 mV) as previously reported [26,32]. UC@CB[7] also showed positive (21 ± 1 mV) zeta potential values.

### 3.2. Contamination with Bacteria/Endotoxin 

**Figure 2:**
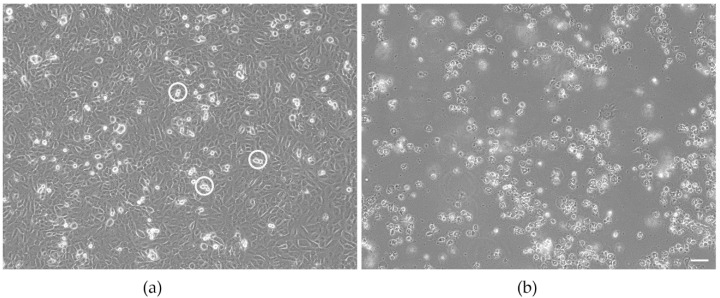
Morphology of untreated EAhy 926 endothelial cells (**a**) and cells exposed to the positive control 20 nm plain polystyrene particles (**b**) after 24 h of culture. Cultures of the untreated EAhy 926 endothelial cells have formed a confluent monolayer with occasional dividing cells (indicated by circles). Cells exposed to the positive control do not form a monolayer but have rounded up, detached from the growth substrate, and float in the medium. Scale bar: 50 µm.

Contamination of samples with living bacteria can easily be detected by microscopic evaluation. The presence of the bacteria induces rapid and not dose-dependent cell death and is, therefore, relatively easy to identify. This was not seen in the UCNP samples tested in this study. Cells exposed to the samples resembled EAhy 926 cells of the growth control (Figure 2a). They formed a confluent monolayer of adherent cells. Cell divisions were seen occasionally.

Dead bacteria usually do not cause acute cell death. However, the lipopolysaccharide complex associated with the outer membrane of Gram-negative pathogen, also termed endoxin, is a strong fever-inducing (pyrogenic) agent [33]. The LAL assay ensures injectable therapeutics are safe for human use determining that the product is endotoxin-free. Endotoxin catalyzes the activation of a proenzyme in the LAL reagent. The activated enzyme (coagulase) hydrolyzes specific bonds within a clotting protein (coagulogen) also present in LAL. Once hydrolyzed, the resultant coagulin self-associates and forms a gelatinous clot. No clot formation was observed, thus indicating the product does not contain free endotoxin (Table 1).

### 3.3. Cytotoxicity

The range of testing was restricted to 200 μg/mL because such concentrations are higher than expected in humans. The dose range allows comparison to the cytotoxicity of superparamagnetic iron oxide nanoparticles, which are in clinical use for magnetic resonance imaging and show no adverse effects up to 100 μg/mL [34].

The first indication of cytotoxicity in EAhy 926 cells, caused either by contamination with bacteria or by exposure to toxicants, is cell detached from the growth substrate, which could be identified by microscopic evaluation. Such changes were only observed in the cultures exposed to the positive control (Figure 2b). 

**Figure 3:**
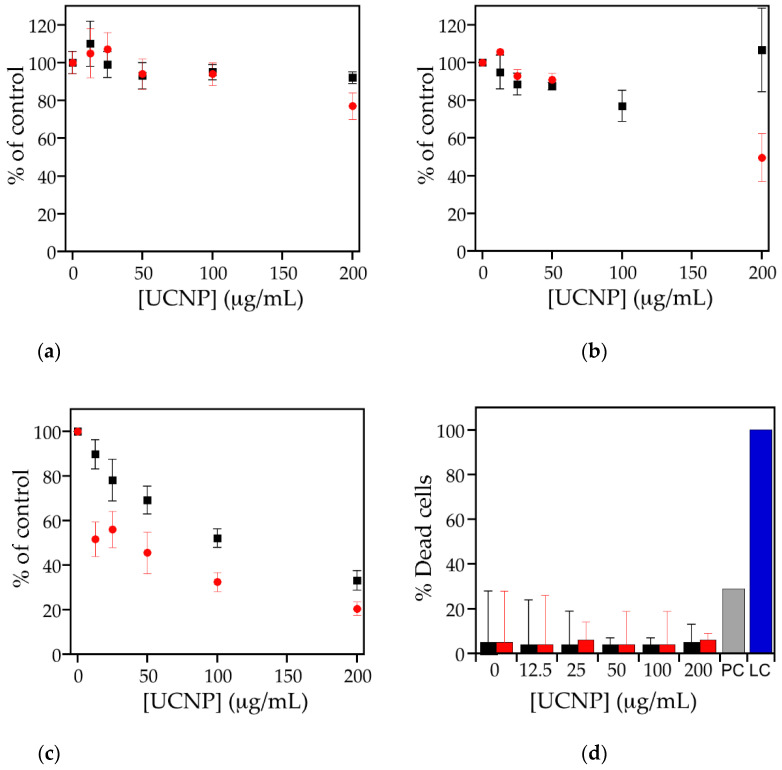
Cell viability (%) of (**a**) EAhy926 endothelial cells, (**b**) HeLa, and (**c**) RAW 264.7 cells according to dehydrogenase activity after incubation with different concentrations of UC@CB[7] (black squares) and UC_n_ (red circles). Cells without exposure to nanoparticles represent 100%. (**d**) LDH release after exposure of EAhy926 cells to different concentrations of UCNPs for 24h. UC@CB[7] (black bar), UC_n_ (red bar), positive control (PC, grey bar), and lysis control (LC, blue bar). Values are shown as mean ± standard deviation.

For a more quantitative analysis of cellular adverse effects, cell-based assays can be used. The most common assay is the determination of dehydrogenase activity using tetrazolium salts. The absorbance is proportional to the number of living cells and suboptimal growth conditions are reflected by decreased activity. In this study, the MTS assay was used. Since particles by themselves may cause absorbance or react with the assay compounds, particles alone (in the absence of cells) were also tested [35]. No action of the UCNPs alone on the absorbance was detected, which means that the UCNPs by themselves do not affect the assay readout. Then, 20 nm non-functionalized polystyrene particles were used as positive control. This particle concentration does not kill all cells and enables us to verify that the cells react as expected. In contrast to cell lysis, which may also serve as positive control, subtle changes in the reaction of the cells can be identified. Cells exposed to the positive control showed 4% viability, which is in the range of the historic values for these particles (2–6%) and indicates that the cells reacted as expected to a toxic stimulus (Figure 3a). According to the definition of cytotoxicity in the ISO10993-5 guideline for medical devices, a decrease of viability >70% compared to untreated or solvent-treated cells defined as 100% is regarded as cytotoxic [36]. Based on this definition, UC@CB[7] and UC_n_ did not show any indication for cytotoxicity in EAhy 926 cells (Figure 3a). 

For verification of the results, LDH activity was determined in the supernatant of the exposed cells. Since nanoparticles are highly reactive due to their large surface, damage of the plasma cell membrane is a common mode of cytotoxic action [37]. If the plasma membrane is disrupted, the intracellularly located enzyme is released from the cells and can be detected in the supernatant. For normalization, the signal obtained after cell lysis is defined as 100% dead cells. In the positive control, 29% of dead cells were detected (data not shown). Compared to that, the amount of cell death in the UCNP- exposed cells with 4–8% was much lower and in the same range as the untreated cells (Figure 3b).

Viability assays on HeLa and RAW 267.4 cell lines performed using 3-(4,5-dimethylthiazol-2-yl)-2,5-diphenyltetrazolium bromide (MTT) assay showed higher viability than 50% at the UCNP concentration used for imaging experiments in both cell lines (50 µg/mL). Nevertheless, cytotoxicity on HeLa and RAW 267.4 depended on the UCNPs tested. UC_n_ and UC@CB[7] showed moderate and low cytotoxicity, respectively, in macrophages in a similar trend to that observed by Kembuan et al. [25], indicating the enhancement of the biocompatibility of UCNPs nanoparticles upon cucurbit[7]uril coating. UC_n_ and UC@CB[7] showed very low cytotoxicity on HeLa; see Figure 3. The higher cytotoxicity observed in HeLa cells compared to the EAhy 926 endothelial cells can be explained by the higher content of lysosomes of the HeLa cells [38]. Since UCNPs can be dissolved in an acidic environment, cells with a higher lysosome content are expected to lead to higher levels of F^–^, Na^+^, Y^3+^, and Ln^3+^ ions with potential cytotoxic effects. UC_n_ were likely to dissolve faster than UC@CB[7]. RAW264.7 cells are professional phagocytes and combine higher content of lysosomes and more efficient uptake of particles than epithelial cells [39]. This may explain the markedly higher cytotoxicity seen in this cell line. 

**Figure 4:**
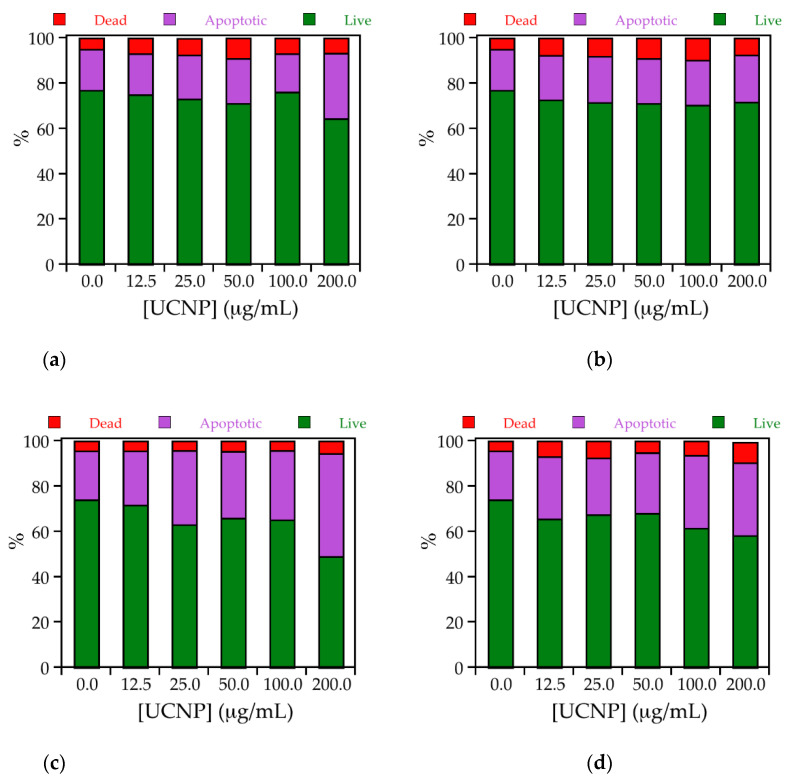
Stack column graph representing the percentages of live (green), dead (red), and apoptotic (purple) cells for treated (top) HeLa and (bottom) RAW 264.7 cells in the presence of 0, 12.5, 25.0, 50.0, 100.0, and 200.0 μg/mL of (**a** and **c**, respectively) UC_n_ or (**b** and **d**, respectively) UC@CB[7].

Calcein AM/ethidium homodimer-1 staining has been used to obtain more information on the dominant mode of cell death induced by the particles. The relatively low number of live cells already in the untreated cells most probably is induced by the detachment of the adherent growing Hela and RAW264.7 cells required for analysis by flow cytometry (Figure 4). Induction of apoptosis was identified as the main mode of cell death with 21–45% in RAW 264.7 cells and with 18–29% in HeLa cells. The rate of apoptosis was higher in RAW 264.7 than in HeLa cells and UC_n_ induced more apoptosis than UC@CB[7] particles. The percentage of 4–10% dead cells was similar in RAW264.7 and HeLa cells and did not differ between UC_n_ and UC@CB[7]. These values are in the same order as measured according to LDH release (Figure 3d). 

**Figure 5:**
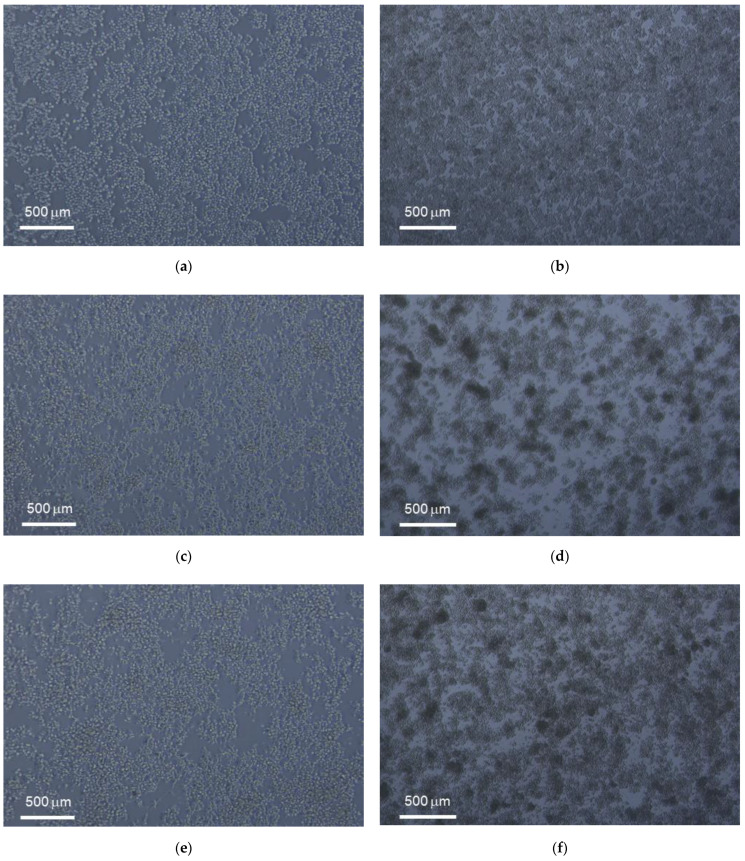
Morphology of (left) HeLa and (right) RAW 264.7 cells exposed to 50 µg/mL after 24 h of culture with untreated, UC_n_ and UC@CB[7] nanoparticles (**a**,**c**,**e** and **b**,**d**,**f**, respectively).

**Figure 6:**
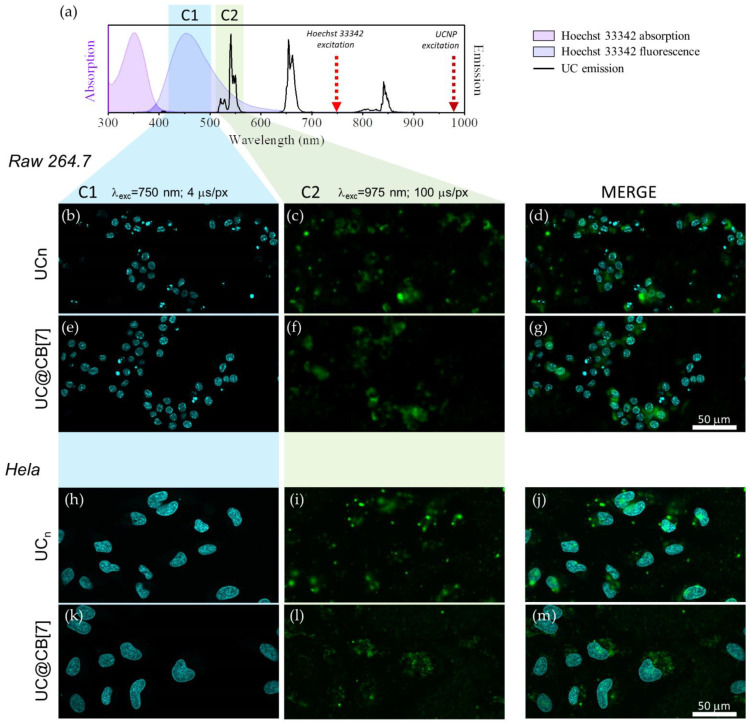
(**a**) Absorption (pink area) and fluorescence (purple area) of Hoechst 33342, emission of UC_n_ dispersion in DMSO (black line) together with the wavelength used to excite the dye/UCNP (arrows) and the microscope detection channels (coloured rectangles). (**b**–**g**) microscope images of RAW 264.7 cells exposed to UC_n_ and UC@CB[7]. (**h**–**m**) microscope images of HeLa cells exposed to UC_n_ and UC@CB[7]. Left column shows the fluorescence signal of Hoechst 33342 (λ_ex_ = 750 nm, dwell time: 4 μs/pixel, detection channel: C1) central column shows the 520/540 nm Er^3+^ upconversion emission (λ_ex_ = 975 nm, dwell time: 100 μs/pixel, detection channel: C2) and right column is a composite of the in line previous images. 50 μm scale bar applies for all images.

Live and fixed cell imaging experiments were conducted for UC_n_, UC@CB[7] in HeLa and RAW 267.4 cell lines. Figure 5 shows the morphology of HeLa and RAW 264.7 cells exposed to 50 μg/mL of UC_n_ and UC@CB[7] after 24 h of culture as compared to untreated cells. The nuclear staining with Hoechst 33342 enables their excitation through two-photon absorption at 750 nm (Figure 6). UCNP imaging was performed exciting the Yb^3+^ ions of the UCNP at 975 nm in the same area imaged previously with Hoechst. UCNP imaging has to be performed under long dwell times (slow scanning speed) in order to avoid the characteristic delay of the long-lived lanthanide emitters which spread its luminescence several pixels away in the scanning direction if the scanning speed is not slow enough [29]. In any case, as it is observed, the UCNP images display some blurriness because a conventional multiphoton microscope UCNP imaging is not confocal since the microscope has not a pinhole before the direct detection to avoid the out-of-focus luminescence and that the upconversion phenomenon, rather than being a two-photon absorption process, is a single photon absorption (Yb^3+^ excitation occurs at 975 nm) followed by several energy transfer processes (from excited Yb^3+^ ions to Er^3+^ ions in the UCNP) [29]. The overlapping of previous images of the same area clearly showed the UCNP cellular internalization in macrophages and cancer cell lines.

Viability assays performed using the MTT assay showed higher viability than 50% at the UCNP concentration used for imaging experiments in both cell lines (50 µg/mL). All UCNPs exhibited a green fluorescence emission located in the cytoplasmatic cell compartment upon excitation at 975 nm with a characteristic lifetime delay on each image pixel. The nuclear staining with Hoechst 33342 showed the nucleus. The imaging assays clearly showed the UCNP cellular internalization in macrophages and cancer cell lines.

## 4. Discussion

CB[7]-capped UCNPs were synthesized by following a previously described protocol [21]. CB[7] was selected to cover the NPs due to its water-solubility, biocompatibility, and ability to interact with many ions and organic molecules via the carbonyl portals. The synthesized nanohybrids were characterized by HRTEM. In comparison to the bare UCNPs, no changes in the NP size or shape were observed after their covering with CB[7]. In Figure 1b, we can clearly distinguish an organic layer surrounding the inorganic matrix with an estimated thickness of ca. 1.5 nm. This thickness can be attributed to a single layer of CB[7] as previously described (height of CB[7] ~ 0.91 nm[40]), thus corroborating the presence of the CB[7] on the NP surface. 

The positive zeta potential values (21 ± 1 mV) of the UC@CB[7] nanohybrids dispersed in milliQ-water are in accordance with the presence of CB[7] as a capping as reported for other CB[7]-capped nanoparticles [41,42].

Moreover, the recorded steady-state photoluminescence spectrum of the UC@CB[7] upon 975-nm excitation displayed the typical sharp emission bands of the Er^3+^ cations, i.e., ca. 520 nm (^2^H_11/2_, ^4^S_3/2_ → ^4^I_15/2_), 540 nm (^4^S_3/2_ → ^4^I_15/2_), and 660 nm (^4^F_9/2_ → ^4^I_15/2_) [43]. 

These assays determined that UC@CB[7] nanohybrids present low cytotoxicity and that more than 90% of the cells remained alive even when using high UC@CB[7] concentrations (200 μg/mL). 

NaYF_4_-based materials in aqueous environments under high dilution conditions can release fluoride and lanthanide ions, which are cytotoxic [22]. It has been reported that fluoride concentrations of 120 ppm decreased cell viability up to 60% [44]. Oleic acid may also contribute to cytotoxicity. Using human aortic endothelial cells, dehydrogenase activity decreased significantly when exposed to polyethylene glycol (PEG)-oleate capped when compared to bare NaGdF_4_:Yb, Er nanoparticles [45]. The bare UCNPs in that study decreased viability to ~70% at a concentration of 75 µg/mL, whereas viability in cells treated with PEG-oleate capped UCNPs was 50% after exposure to 5 µg/mL. The study demonstrated that dissociation of the PEG-oleate group from the nanoparticles was the main factor for cytotoxicity. In the present study, oleate was removed prior to functionalization with CB[7], consequently the nanoparticles were more biocompatible than PEG-oleate capped UCNPs. Phenylalanine, lysine, and glucose, which are main components of the cell culture medium Dulbecco’s Modified Eagle Medium (DMEM) used in this study, have been shown to adsorb on the surface of the UCNP and stabilize the nanoparticles [46]. This effect most probably also contributes to the absence of cytotoxicity of the UCNPs used in this study. Amino acids and glucose are also components of blood and many other physiological fluids. The study by Ju et al. 2017 showed that the low cytotoxicity in vitro corresponds to lack of adverse effects after intravenous injection of polyethyleneimine (PEI)-UCNPs into mice [47]. 

For the assessment of cytotoxic effects, cells relevant for the commonly used intravenous administration of UCNPs were selected, namely EAhy 926 endothelial cells as the lining cells of the blood vessels, RAW 264.7 as models for professional phagocytes (monocytes in the blood) and HeLa cells for epithelial cells, which are the main components of the parenchyma. Cell lines, instead of primary cells, were used because assessment of basal cytotoxicity, and not of specific cell function, was intended. For this type of testing, the reproducibility of cell lines is a major advantage [36]. Standardized testing of nanomaterials also includes the verification of the absence of endotoxin, which, in this study, showed that particles were free of contamination with bacterial compounds [48]. The viability assays based on dehydrogenase detection (MTT and MTS assay) indicated that UC_n_ induced greater effects than UC@CB[7]. Higher cytotoxicity was also reported previously for uncoated UCNPs when compared to silica-coated ones in RAW 264.7 cells and was attributed to higher internalization of negatively charged uncoated UCNPs [25]. Similarly, our results also indicate that a particle concentration up to 12.5 μg/mL does not lead to a critical cell line viability.

Combination of multiple endpoints is suggested, particularly for the assessment of nanomaterials [49]. In this study, the combination of microscopy, dehydrogenase content, calcein AM/ethidium homodimer-1 staining and LDH release was used. The lack of increased LDH release compared to the controls indicates that plasma membrane damage is not the mechanism of cytotoxic action caused by the UC_n._ Calcein AM/ethidium homodimer-1 staining confirmed this finding and revealed induction of apoptosis as main mode of cytotoxic action. The same mode of toxic action has also been reported for other UCPNs, for instance NaGdF_4_:Yb^3+^, Er^3+^ particles NaGdF_4_ [24]. 

A limitation of the study is the fact that cytotoxicity was not tested upon illumination. However, since near-infrared light used for irradiation is biocompatible and UCNPs do not warm up when irradiated, no additional cytotoxic effect is expected. The study is further limited by the fact that the monocultures lack the complexity of the organism and not all aspects of toxicity have been covered. Therefore, further studies to assess hemocompatibility in blood, genotoxicity, immunogenicity, and cell-specific toxicity are needed. Nevertheless, this initial biological testing identified UC_n_ and UC@CB[7] nanohybrids as promising nanomaterials that warrant further testing. 

## Figures and Tables

**Table 1:** Results of the LAL assays at different concentrations of UCNPs in duplicate.

Nanomaterial	Concentration/μg/mL	Clot Test 1 ^1^	Clot Test 2 ^1^
UC_n_	200	-	-
	20	-	-
	2	-	-
	5	-	-
UC@CB[7]	200	-	-
	20	-	-
	2	-	-
	5	-	-

^1^ (-): no clot formation.

## Data Availability

The data presented in this study are available in the article.
